# Peripheral nerve block combined with general anesthesia for lower extremity amputation in hemodialysis patients: case series

**DOI:** 10.1186/s40981-018-0214-x

**Published:** 2018-10-25

**Authors:** Hitomi Takemura, Daisuke Fujita, Megumi Matsuda, Kazuko Fujita, Masahiro Sakaguchi, Fumimasa Amaya

**Affiliations:** 1 Department of Anesthesiology, Kyoto Prefecture University of Medicine, Kajiicho 465, Kamigyo-ku, Kyoto, 602-8566 Japan; 2 Department of Anesthesiology, Japanese Red Cross Kyoto Daiichi Hospital, 15-749 Motomachi, Higashiyama-ku, Kyoto, 605-0981 Japan; 3 Department of Anesthesiology, Center for Translational Pain Medicine, Duke University Medical Center, Durham, NC 27710 USA; 4 Department of Anesthesiology, Kansai Medical Hospital, 1-1-7-2 Shinsenri, Nishimachi, Toyonaka, Osaka 560-0083 Japan

**Keywords:** Peripheral nerve block, Hemodialysis patients, Lower extremity amputation

## Abstract

**Background:**

Anesthetic management of lower extremity amputation in chronic hemodialysis (HD) patients can be challenging because of their poor cardiovascular status. As previously reported, peripheral nerve block (PNB) may be beneficial in these complicated cases. We report the effects of PNB combined with general anesthesia on hemodynamic stability in HD patients undergoing elective lower extremity amputation.

**Methods:**

We retrospectively analyzed 13 HD patients who underwent lower extremity amputation. Patients received general anesthesia (GA group, *n* = 7) or general anesthesia combined with PNB (GA with PNB group, *n* = 6), as decided by the anesthesiologists. Mean blood pressure (MBP), systolic blood pressure (SBP), lowest BP, heart rate (HR), blood loss, fluid and blood infusion volumes, and doses of vasopressors required were compared for hemodynamic assessment. The coefficient of variation ($\mathrm{CV}=\upsigma /\overline{\mathcal{X}}$) of MBP (CV_MBP_) and SBP (CV_SBP_) was calculated to compare hemodynamic stability. Intraoperative opioid use and postoperative pain scores at rest using a numerical rating scale (NRS) on postoperative days 0 and 1 were compared for pain assessment. We also assessed 30-day mortality.

**Results:**

CV_MBP_ in the GA group was significantly higher than that in the GA with PNB group (0.15 ± 0.05 and 0.08 ± 0.04, respectively, *p* = 0.03). The CV_SBP_ in the GA group was also significantly higher than that in the GA with PNB group (0.16 ± 0.02 and 0.09 ± 0.01, respectively, *p* = 0.03). No significant differences in other hemodynamic parameters were observed. Intraoperative fentanyl doses were significantly lower in the GA with PNB group (GA 210.7 ± 99.9 μg vs. GA with PNB 113.0 ± 75.6 μg, *p* = 0.04). There were no significant differences in other pain parameters and 30-day mortality between the groups.

**Conclusion:**

Our results suggest that PNB combined with general anesthesia contributes to intraoperative hemodynamic stability through better pain control in HD patients undergoing lower extremity amputation.

## Introduction

Peripheral artery disease is recognized as a serious complication in patients requiring chronic hemodialysis (HD). The anesthetic management of lower extremity amputation for critical limb ischemia, an advanced form of peripheral artery disease, in HD patients can be challenging because of their poor cardiovascular status, including blood pressure instability [1]. As previously reported, peripheral nerve block (PNB) may be beneficial in these complicated cases [2, 3].

The coefficient of variation (CV), the ratio of the standard deviation to the mean, is widely used to represent the degree of variation of data. Hemodynamic instability, indicated by higher values of the CV of blood pressure during surgery, is associated with poor prognosis [4]. However, no study has described the relationship between anesthetic management with PNB and CV value of blood pressure. We retrospectively reviewed the cardiovascular status, including the CV value of blood pressure, in HD patients during lower extremity amputation under general anesthesia with or without PNB.

## Methods

Eligible patients included chronic HD patients who underwent lower extremity amputation between November 2014 and December 2015 at the Japanese Red Cross Kyoto Daiichi Hospital, Kyoto, Japan. The study protocol was approved by the ethics committee of the Japanese Red Cross Kyoto Daiichi Hospital (reference no. 498). Inclusion criteria were HD patients undergoing elective lower extremity amputation under general anesthesia or general anesthesia combined with PNB. Exclusion criteria were pacemaker implantation prior to the surgery, inability to extubate the patient in the operating room, and patients younger than 20 years old. The sample size was determined based on the number of lower extremity amputations in hemodialysis patients performed at our hospital during the study period.

Patient characteristics, surgical procedures, anesthetic method, blood loss volume, fluid and blood infusion volumes, dose of vasopressor, opioid requirements during the surgery, pain intensity after the surgery measured using a numerical rating scale (NRS), and prognosis 30 days after the surgery were retrospectively recorded from the patients’ medical charts. Data on mean blood pressure (MBP), systolic blood pressure (SBP), and heart rate (HR) recorded at 5-min intervals during the surgery were also collected. Information on the nerve block procedure and amount of local anesthetic were also recorded for patients who received PNB.

Since this study was conducted retrospectively, perioperative management, choice of anesthetic method, and cardiovascular management were decided by the attending anesthesiologist. General anesthesia was maintained with 1.5–2% sevoflurane, remifentanil, fentanyl, and rocuronium. Ephedrine, phenylephrine, or dopamine was used to treat hypotension. The radial artery was cannulated for blood pressure measurement in all patients. PNB was performed prior to the surgery under ultrasound and nerve stimulator guidance with levobupivacaine (0.25–0.375%, 15–45 mL). In some cases, PNB was performed before the induction of general anesthesia. Postsurgical pain intensity was assessed during an interview conducted by the attending anesthesiologist on the day of surgery and 1 day after the surgery. Pain intensity at rest was measured on a NRS. The CV of hemodynamic variables, defined by the formula, $\mathrm{CV}=\upsigma /\overline{\mathcal{X}}$ (standard deviation/arithmetic mean), was calculated to compare hemodynamic stability.

Patients were divided into two groups based on the anesthetic procedure: those who received general anesthesia (GA group, *n* = 7) and those who received PNB combined with general anesthesia (GA with PNB group, *n* = 6). The main outcome analyzed in this study was the CV of MBP (CV_MBP_) in the two groups. The CV of SBP (CV_SBP_), the CV of HR (CV_HR_), average and lowest MBP during the surgery, 30-day mortality, vasopressor and opioid requirements during the surgery, and postoperative pain scores were compared as secondary outcomes. Chronological MBP changes relative to the initial value were calculated by the formula:

$$ \mathrm{Relative}\ \mathrm{change}\ \mathrm{in}\ \mathrm{MBP}\ \left(\%\right)\kern0.75em =\kern0.5em \ \left\{\left(\mathrm{MBP}-\mathrm{initial}\ \mathrm{MBP}\right)/\mathrm{initial}\ \mathrm{MBP}\right\}\times \kern0.37em 100 $$

Initial MBP was defined as MBP measured in the operating room immediately after the arrival. Relative changes in MBP were compared between the two groups.

Data was analyzed using the *t* test, Mann-Whitney *U* test, or chi-square test, as appropriate, using GraphPad Prism software (Ver.7.00, GraphPad Software, San Diego, CA USA). We considered *p* < 0.05 to indicate statistical significance.

## Results

**Fig. 1:**
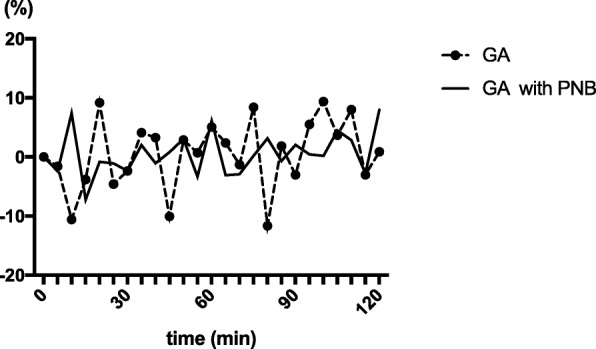
Chronological changes in MBP. Chronological MBP changes relative to the initial value were calculated by the formula: Relative change in MBP (%) = {(MBP − initial MBP)/initial MBP} × 100, initial MBP was defined as MBP measured in the operating room immediately after arrival

The demographic data of the participants are shown in Table 1. There were no significant differences in the patients’ background characteristics between the two groups. The CV_MBP_ in the GA group was significantly higher than that in the GA with PNB group (0.15 ± 0.05 and 0.08 ± 0.04, respectively, *p* = 0.03). The CV_SBP_ in the GA group was also significantly higher than that in the GA with PNB group (0.16 ± 0.02 and 0.09 ± 0.01, respectively, *p* = 0.03). The CV_HR_ and average and lowest MBP were not statistically significantly different between the two groups (Table 2). The mortality rate 30 days after the surgery was similar between the two groups. Ephedrine and/or phenylephrine was required to treat low blood pressure in five (71.4%) and five (83.3%) of the GA group and GA with PNB group, respectively. One patient in the GA with PNB group required a continuous intravenous infusion of dopamine (3–7 μg/kg/min) for sustained low blood pressure. Blood loss volumes, fluid and blood infusion volumes, and the doses of vasopressors were also not different between the two groups. Fentanyl was used in seven (100%) and four (66.7%) of the GA group and GA with PNB group, respectively. The intraoperative dose of fentanyl was significantly lower in the GA with PNB group (GA 210.7 ± 99.9 μg vs. GA with PNB 113.0 ± 75.6 μg, *p* = 0.04, Table 2). Remifentanil was used in seven (100%) and five (83.3%), respectively. Intraoperative remifentanil dose was not significantly different between the two groups (GA 0.5 ± 0.23 mg vs. GA with PNB 0.6 ± 0.35 mg, *p* = 0.51, Table 2). There were no significant differences in postoperative pain scores between the groups. Relative changes in MBP were relatively smaller in the GA with PNB group compared to the GA group (Fig. 1).

**Table 1:** Characteristics of the patients in each group

	GA (*n* = 7)	GA with PNB (*n* = 6)	*P* value
Age (years)	72.6 ± 3.9	70.7 ± 2.0	0.20
Sex (male/female)	5/2	3/3	0.42
Height (cm)	163.0 ± 8.2	159.2 ± 5.4	0.36
Weight (kg)	60.8 ± 15.9	58.0 ± 7.3	0.54
ASA-PS			
III	6 (85.7%)	5 (83.3%)	
IV	1 (14.3%)	1 (16.7%)	
Medical history			
DM	6	5	
IHD	6	4	
CI	1	3	
Af	1	1	
HT	0	1	
Surgical procedures			
BKA	5	3	
AKA	1	3	
TA	1	0	
Duration of surgery (min)	111.2 ± 24.2	104.5 ± 32.1	0.67
Duration of anesthesia (min)	209.1 ± 31.6	227.5 ± 50.4	0.44
Initial MBP (mmHg)	86.6 ± 28.9	74.6 ± 8.3	0.36
Initial HR (bpm)	79.3 ± 18.6	76.7 ± 22.6	0.73

*ASA-PS* American Society of Anesthesiologists (ASA) Physical Status, *AKA* above-knee amputation, *BKA* below-knee amputation, *TA* toes amputation, *DM* diabetes mellitus, *IHD* ischemia heart disease, *HT* hypertension, *CI* cerebral infarction, *Af* atrial fibrillation, *MBP* mean blood pressure, *HR* heart rate

**Table 2:** Study results

	GA	GA with PNB	*P* value
CV of MBP	0.15 ± 0.05	0.08 ± 0.04	0.03^†^
CV of SBP	0.16 ± 0.02	0.09 ± 0.01	0.03^†^
CV of HR	0.08 ± 0.02	0.06 ± 0.02	0.46
Averaged MBP (mmHg)	72.3 ± 13.1	69.37 ± 7.26	0.65
Lowest MBP (mmHg)	56.7 ± 7.9	59.1 ± 7.8	0.58
Fentanyl (μg)	210.7 ± 99.9	113.0 ± 75.6	0.04^†^
Remifentanil (mg)	0.5 ± 0.23	0.6 ± 0.35	0.51
Ephedrine (mg)	4.0 ± 4.0	10 ± 14.3	0.77
Phenylephrine (mg)	0.27 ± 0.48	0.20 ± 0.19	0.55
Transfusion (ml)	835.7 ± 343.6	766.7 ± 267.7	0.67
Blood transfusion (ml)	155.7 ± 211.3	130.0 ± 142.8	0.86
Blood loss (ml)	138.8 ± 147.1	171.7 ± 189.8	0.72
NRS (POD0)	4.5 ± 4.8	0.5 ± 0.58	0.40
NRS (POD1)	4.3 ± 3.9	1.5 ± 1.7	0.28
30-day Mortality (%) (*n*)	14.3 (1)	16.7 (1)	0.91

*MBP* mean blood pressure, *NRS* numerical rating scale, *POD* postoperative day, *CV* coefficient of variation

† : *P* < 0.05

No patient developed PNB-related complications, such as hematoma, paralysis, or local anesthetic intoxication.

## Discussion

In the present study, we observed lower BP variability under PNB combined with general anesthesia during leg amputation in patients on chronic HD. Higher intraoperative BP variability is associated with postoperative delirium [5], increased blood loss during the surgery [6], and postoperative mortality [7]. To the best of our knowledge, no previous studies have focused on the effects of PNB combined with general anesthesia for lower extremity amputation in HD patients.

We consider the difference in CV_MBP_ and CV_SBP_ between general anesthesia with or without PNB observed in our study (0.15 vs. 0.08 and 0.16 vs. 0.09, respectively) as being clinically meaningful based on a previous report that considered a smaller difference in CV_BP_ between survivors and non-survivors after cardiac surgery (0.21 vs. 0.24) as being significant [4]. Based on their data, the authors of the previous study concluded that every increase of 0.1 in CV_BP_ was associated with a 150% increase in the risk of death. The prevalence of peripheral artery disease in patients on chronic HD is substantially high [8], and mortality rates after lower leg amputation for critical limb ischemia in HD patients are reportedly as high as 17% at 30 days and 44% at 1 year, with cardiac disease being the leading cause of death [9].

Some authors have reported the successful use of PNB for lower extremity amputation patients, in order to avoid the systemic adverse effects of general anesthesia [2, 3]. Favorable aspects of PNB, such as better pain control and functional recovery, have been emphasized, although the impact of anesthesia methods on postoperative mortality remains controversial [10–12].

Previous studies have indicated the relationship between intraoperative hypotension with postoperative myocardial, kidney injury, or increased mortality [13, 14]. Furthermore, as reported with high-risk abdominal surgery, PNB may contribute to hemodynamic stability when combined with general anesthesia [15]. In the current study, although no significant difference in 30-day mortality was observed between the groups, intraoperative hemodynamic stability without severe hypotension secondary to PNB may lead to favorable outcomes compared with general anesthesia alone.

In this study, it remained unresolved whether hemodynamic instability in the GA group was the nature of opioid-based analgesia or consequent of insufficient analgesia during the surgery. We could not investigate the effect of larger doses of remifentanil on hemodynamic stability since remifentanil doses were similar between the GA and GA with PNB groups. A previous study found lower mean blood pressures despite larger doses of ephedrine in patients who received remifentanil-based analgesia compared to GA with PNB [11].

This study has several limitations. This was a retrospective study conducted at a single hospital. Further, we could not adjust for the other perioperative factors that might have influenced hemodynamic stability, such as anesthetic method, dose of anesthetic drugs, fluid status, surgical procedure, patient age, and cardiac function due to the small sample size. The sample size was limited since we were unable to expand the study period in order to avoid the risk of increasing the heterogeneity of participant background, surgical procedure, and perioperative management. Further, postoperative delirium and phantom pain were not assessed in the study.

## Conclusion

Our results suggest that PNB combined with general anesthesia contributes to intraoperative hemodynamic stability in HD patients who undergo lower extremity amputation.

## Abbreviations

CV: Coefficients of variation
HD: Hemodialysis
HR: Heart rate
MBP: Mean blood pressure
NRS: Numerical rating scale
PNB: Peripheral nerve block
SBP: Systolic blood pressure

## References

[1] Chirakarnjanakorn S, Navaneethan SD, Francis GS, Tang WH (2017). Cardiovascular impact in patients undergoing maintenance hemodialysis: clinical management considerations. Int J Cardiol.

[2] Bech B, Melchiors J, Borglum J, Jensen K (2009). The successful use of peripheral nerve blocks for femoral amputation. Acta Anaesthesiol Scand.

[3] Baddoo H (2009). A preliminary report on the use of peripheral nerve blocks for lower limb amputations. Ghana Med.

[4] Jinadasa SP, Mueller A, Prasad V, Subramaniam K, Heldt T, Novack V (2018). Blood pressure coefficient of variation and its association with cardiac surgical outcomes. Anesth Analg.

[5] Hirsch J, DePalma G, Tsai TT, Sands LP, Leung JM (2015). Impact of intraoperative hypotension and blood pressure fluctuations on early postoperative delirium after non-cardiac surgery. Br J Anaesth.

[6] Matsuura N, Okamura T, Ide S, Ichinohe T (2017). Remifentanil reduces blood loss during orthognathic surgery. Anesthesia Progress.

[7] EJ M, D Y SW, DI S (2015). Intraoperative mean arterial pressure variability and 30-day mortality in patients having noncardiac surgery. Anesthesiology.

[8] Cheung AK, Sarnak MJ, Yan G, Dwyer JT, Heyka RJ, Rocco MV (2000). Atherosclerotic cardiovascular disease risks in chronic hemodialysis patients. Kidney Int.

[9] Serizawa F, Sasaki S, Fujishima S, Akamatsu D, Goto H, Amada N (2016). Mortality rates and walking ability transition after lower limb major amputation in hemodialysis patients. J Vasc Surg.

[10] Khan SA, Qianyi RL, Liu C, Ng EL, Fook-Chong S, Tan MG (2013). Effect of anaesthetic technique on mortality following major lower extremity amputation: a propensity score-matched observational study. Anaesthesia.

[11] Kim NY, Lee KY, Bai SJ, Hong JH, Lee J, Park JM (2016). Comparison of the effects of remifentanil-based general anesthesia and popliteal nerve block on postoperative pain and hemodynamic stability in diabetic patients undergoing distal foot amputation: a retrospective observational study. Medicine (Baltimore).

[12] Scott SW, Bowrey S, Clarke D, Choke E, Bown MJ, Thompson JP (2014). Factors influencing short- and long-term mortality after lower limb amputation. Anaesthesia.

[13] Monk TG, Bronsert MR, Henderson WG, Mangione MP, Sum-Ping ST, Bentt DR (2015). Association between intraoperative hypotension and hypertension and 30-day postoperative mortality in noncardiac surgery. Anesthesiology.

[14] van Waes JA, van Klei WA, Wijeysundera DN, van Wolfswinkel L, Lindsay TF, Beattie WS (2016). Association between intraoperative hypotension and myocardial injury after vascular surgery. Anesthesiology.

[15] Tsuchiya M, Takahashi R, Furukawa A, Suehiro K, Mizutani K, Nishikawa K (2012). Transversus abdominis plane block in combination with general anesthesia provides better intraoperative hemodynamic control and quicker recovery than general anesthesia alone in high-risk abdominal surgery patients. Minerva Anestesiol.

